# Dynamics of Two Multi-Stemmed Understory Shrubs in Two Temperate Forests

**DOI:** 10.1371/journal.pone.0098200

**Published:** 2014-06-02

**Authors:** Xuejiao Bai, Tania Brenes-Arguedas, Ji Ye, Xugao Wang, Fei Lin, Zuoqiang Yuan, Shuai Shi, Dingliang Xing, Zhanqing Hao

**Affiliations:** 1 College of Forestry, Shenyang Agricultural University, Shenyang, Liaoning Province, China; 2 State Key Laboratory of Forest and Soil Ecology, Institute of Applied Ecology, Chinese Academy of Sciences, Shenyang, Liaoning Province, China; 3 Centro Cientifico Tropical, San Jose, Costa Rica; 4 University of Chinese Academy of Sciences, Beijing, China; DOE Pacific Northwest National Laboratory, United States of America

## Abstract

A multi-stemmed growth form may be an important trait enabling the persistence of individual shrubs in the forest understory. With the aim of evaluating the role of multiple stems, neighbor competition and soil nutrients in shrub performance, we study the dynamics of two temperate multi-stemmed shrub species. We modeled stem growth and survival of *Corylus mandshurica* and *Acer barbinerve* in two temperate forests with differing structure in northeastern China. One forest was an old growth broad-leaved Korean pine (*Pinus koraiensis*) mixed forest; the other was a secondary poplar-birch forest. Growth of the two species and survival of *C. mandshurica* increased with stem number in the old growth forest, but not the secondary forest, suggesting the benefits of a multi-stemmed growth form are facultative. *C. mandshurica* also suffered more from overstory neighbor competition in the old growth forest, which may suggest that this species is less shade-tolerant than *A. barbinerve*. Moreover, the performance of the two species were clearly influenced by understory neighbors and soil variables in the old growth forest relative to the secondary forest, which may be due to different forest structure. We conclude that multiple stems are not always important for the persistence of shrub species. Even within the same species, the multi-stemmed benefits might be facultative, differing among forests and neighborhood compositions.

## Introduction

While forest studies often focus on tree dynamics, shrubs are also ubiquitous components of the forest community, and their dynamics differ from dominant trees in important ways. Shrubs influence ecosystem properties by maintaining plant diversity, influencing tree seedling regeneration [1], [2], and affecting soil nutrient flow [3]–[5]. However, the demographic traits of understory shrub species are fundamentally different from overstory tall tree species. The dynamics of understory shrubs are mostly driven by vegetative growth and mechanical damage to stems [6]. Also, unlike overstory tall trees, shrubs can spend all their life in the forest understory.

Many shrub species produce multiple stems through vegetative reproduction. The replacement of stems within a shrub has been thought of as an adaptation enabling long persistence of individual shrubs in their environments, as it may allow for increased acquisition of resources in heterogeneous environments [7]. In multi-stemmed shrubs, each stem may experience recruitment, growth, and death semi-independently. Thus, such shrubs usually consist of stems of different ages and sizes [8]. However, interactions among stems in a multi-stemmed plant may be complex. For example, competition among stems within a shrub might restrict height growth relative to single-stemmed trees [9], while more stems can increase overall plant survival compared with individuals of the same species with fewer stems [10].

To understand the dynamics of multi-stemmed understory shrubs, it is important to account for the biotic environment in the understory. Understory shrubs are usually limited by tree competition. Denser neighborhoods may reduce shrub performance due to resource competition. One study that investigated shrub dynamics as a function of the understory neighbor structure suggested that the neighboring understory trees negatively affected the absolute diameter growth and survival of large ramets [11]. Also, large individuals can suppress the performance of smaller individuals [12], as canopy trees usually dominate forest light and root environments [13]. Some researchers have investigated the influence of overstory characteristics on diversity and abundance of shrub species [14], [15]. Soil conditions may also affect the performance of understory shrub species. For example, the reproductive modes of an understory shrub was related to soil environment, and the multi-stemmed growth form was preferred in places with abundant litter substrate [16]. Similarly, the dominance and frequency patterns of multi-stemmed plants were driven by soil nutrient status [17], [18]. In addition, size strongly affected the absolute diameter growth and survival of large ramets for shrub species [11]. Therefore, soil variables and stem size should be also considered to examine the dynamics of multi-stemmed understory shrubs.

During forest development, floristic composition, diversity, richness, and density of plant communities may change [19]. Hence, the existence or strength of biotic processes, such as neighborhood interactions, can vary at different successional stages, particularly if large changes occur in the vertical structure of plant communities through time [15]. Moreover, soil nutrients are more available in old growth forests owing to less efficiency in nutrient retention than secondary stage in some cases [20]. To our knowledge, no ecological research has been conducted in dynamics of understory shrubs in old growth compared with secondary forests.

The goal of this study is to evaluate the role of stem size, multiple stems, neighbor competition and soil conditions in stem demographic variables of two shrub species in two temperate forests at different successional stages. We investigated the effects of diameter at breast height (dbh), the number of stems in a shrub, overstory and understory neighbors and soil variables. We specifically ask the following questions: (1) How does the number of stems in a shrub affect stem growth and survival of these two shrub species? (2) How do basal area of overstory and understory neighbors and soil variables affect stem growth and survival of these two species? And (3) do the effects of multiple stems, neighbors and soil variables differ between the two study forests? We predict that competition from local neighbors is going to be stronger, and have a more negative impact on growth and survival of these two species in the old growth forest, where there are more larger and older trees [21].

## Materials and Methods

### Ethics statement

The study forests represent neither privately-owned field, nor endangered or protected species. No specific permits were required for the described field studies.

### Study site

We studied two forests in northeastern China, both located in Changbai Mountain Nature Reserve (42°23′N, 128°05′E), which is one of the largest biosphere reserves in China and has been spared from logging and other severe human disturbances. The reserve was established in 1960 and joined the World Biosphere Reserve Network under the Man and the Biosphere Project in 1980 [22], [23]. The reserve is about 200,000 ha with elevation ranging from 740 m to 2,691 m [24].

One of our study sites is in broad-leaved Korean pine (*Pinus koraiensis*) mixed temperate forest, which is the dominant vegetation type in northeastern China and well known for high species richness and particular species composition [23], [25]. The other is a secondary poplar-birch forest, which is one of the main secondary forest types on Changbai Mountain, resulting from natural regeneration after clear-cutting or fire. The poplar-birch forest is an important stage in the secondary succession of the broad-leaved Korean pine forest [26]. For brevity, we will hereafter refer to these two forests as the ‘old growth forest’ and the ‘secondary forest’ respectively. The climate of the study region is characterized by low temperature and high precipitation [24]. Mean annual temperature is 3.3°C (−16.5°C in January and 20.5°C in August). Mean annual precipitation is 672 mm, most of which occurs between June and September (480–500 mm) [24].

### Plot censuses

Both these sites have large forest dynamic plots where all individuals with diameter at breast height ≥1 cm have been measured, mapped, tagged, and identified to species. Trees and shrubs are re-measured every five years. In the old forest, the forest dynamics plot is 25 hectare (ha) (500×500 m). It was established in 2004 and recensused in 2009. The elevation in the plot ranges from 791.8 m to 809.5 m. In the 2004 census, there were 59,138 living stems, belonging to 52 species, 34 genera, and 18 families. Mean stand density of living stems was 2,366 stems·ha^−1^ and mean basal area of living trees was 43.75 m^2^·ha^−1^.

In the secondary forest, the forest dynamic plot is 5 ha (250×200 m). It was established in 2005 and recensused in 2009. The elevation ranges from 796.3 m to 800.4 m. In the 2005 census, there were 20,107 living stems, belonging to 50 species, 32 genera, and 18 families. Mean stand density of living stems was 4,021 stems·ha^−1^ and mean basal area of living trees was 28.79 m^2^·ha^−1^. Hence, relative to the old growth forest, in this plot there were much more stems, but these were considerably smaller, suggesting lower canopy height and a denser understory.

### Study species

**Figure 1:**
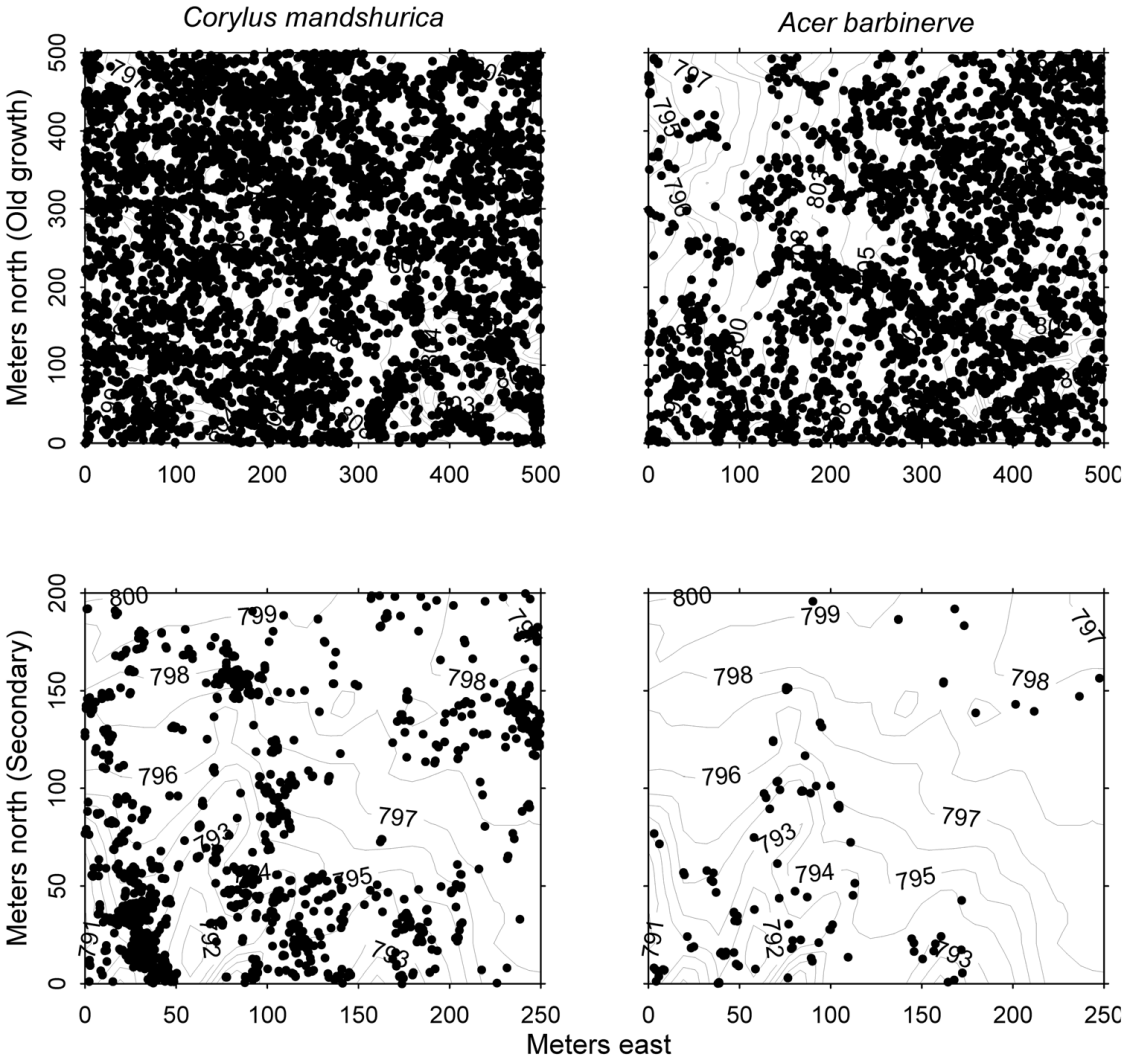
The distribution of *C. mandshurica* and *A. barbinerve* in the old growth and secondary forest plots. The solid points represent the individuals of the two species. The contour lines refer to the topography of the two forest plots.

Here we focus on the stem growth and survival dynamics of two shrub species that are most abundant in the understory of both forest types. The first is *Corylus mandshurica* (English common name Manchurian Filbert), an androgynous shrub with edible fruits that are dispersed by gravity. The second species is *Acer barbinerve* (common name Barbed Vein Maple), a dioecious species with wind dispersed seeds. The distributions of these two shrub species in the two forest plot are shown in Figure 1. A large proportion of individuals of these two species have multiple stems and form clumps [27]. Here, we evaluated the dynamics of stems in these clumps.

### Statistical analysis

To examine the explanatory variables affecting the dynamics of shrub stems, we used generalized linear mixed models (GLMMs), with gamma errors and log-link function for the growth rate, and with binomial errors and logit-link function for the survival probability. The variables in the models are described below.

#### Demographic rates

Growth of each shrub stem was calculated as basal area increment (cm^2^ yr^−1^): 
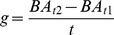
where BA_ti_ is the basal area of shrub stems in census i, and t is the inter-census interval. We excluded extremes from the calculation of all shrub stems decreasing in size by >5% yr^−1^ [28], and altered remaining negative growth rates to be zero. To log transform growth, we added 0.01.

#### Biotic variables

For each shrub, we calculated the following variables: (1) the number of stems in the shrub, (2) basal area of overstory neighbors (dbh≥10 cm) between 5–20 m radii, (3) basal area of understory neighbors (dbh<10 cm) between 5–20 m radii. To eliminate boundary effects, only those shrubs with a distance greater than or equal to 20 m from the forest plot edges were included.

#### Soil variables

We sampled soils within the two forest plots using a regular grid of points every 30 m. At each grid point, two additional sample points at 2, 5 or 15 m were selected in a random compass direction from the grid point. In total, 967 and 215 soil samples were sampled in the old growth and secondary forest plots respectively. Soil properties (organic matter, total nitrogen, total phosphorus and total potassium) showed significant differences among the two forests (organic matter: t = 8.703, *P*<2.2e-16; total nitrogen: t = −4.551, *P* = 7.331e-06; total phosphorus: t = 4.725, *P* = 3.28e-06; total potassium: t = 16.254, *P*<2.2e-16). There were higher content of organic matter, total phosphorus and total potassium, but lower content of total nitrogen in the old growth forest (Mean±S.E.: 165.284±2.646 g·kg^−1^ vs. 129.324±3.173 g·kg^−1^; 1.255±0.015 g·kg^−1^ vs. 1.111±0.027 g·kg^−1^; 16.472±0.086 g·kg^−1^ vs. 13.661±0.150 g·kg^−1^; 6.370±0.087 g·kg^−1^ vs. 7.190±0.158 g·kg^−1^). Soil organic matter did not show spatial autocorrelation in the secondary forest. We interpolated total nitrogen, total phosphorus and total potassium of the two forest plots to 5×5 m grid by kriging. Then, we converted the calculated values for each grid to z scores [29]. To reduce possibility of overfitting the models [29], we performed a principal components analysis (PCA) on these three soil variables. The first two principle components accounted for 98.8% of the total variance in the three soil variables in the old growth forest and 91.8% in the secondary forest (Table S1 in File S1). In both forests, the first component (PC1) explained variation in total nitrogen and phosphorous, while the second (PC2) represented variation in total potassium.

In all models we included dbh (log-transformed), biotic variables and soil variables (Table 1) as explanatory variables. The values of the explanatory variables were standardized by subtracting the mean value of the variable and dividing by the standard deviation. Because shrub stems from the same individual were not independent replicates of stem number, we included individual as a random effect.

**Table 1:** Parameters included in models.

Variables	Data		
	Range	Mean	Median
**Old growth forest**			
***Corylus mandshurica*** ** (13,498)**			
DBH (cm)	1–10.9	1.580	1.5
Number of stems in a shrub	1–18	3.318	3
Basal area (m^2^) of overstory neighbors within 20 m	2.217–10.447	5.340	5.284
Basal area (m^2^) of understory neighbors within 5 m	0–0.044	0.010	0.009
Soil PC1	−2.024–3.279	−0.008	−0.497
Soil PC2	−1.015–1.377	0.140	0.119
***Acer barbinerve*** ** (10,216)**			
DBH (cm)	1–13.2	1.931	1.7
Number of stems in a shrub	1–21	5.394	5
Basal area (m^2^) of overstory neighbors within 20 m	3.066–9.592	5.523	5.470
Basal area (m^2^) of understory neighbors within 5 m	0–0.042	0.011	0.010
Soil PC1	−2.020–3.299	−0.633	−1.066
Soil PC2	−0.946–1.391	0.034	0.013
			
**Secondary forest**			
***Corylus mandshurica*** ** (1,269)**			
DBH (cm)	1–4.9	1.551	1.5
Number of stems in a shrub	1–13	2.940	2
Basal area (m^2^) of overstory neighbors within 20 m	1.705–4.249	3.098	3.180
Basal area (m^2^) of understory neighbors within 5 m	0.000–0.073	0.033	0.032
Soil PC1	−2.576–3.525	0.579	0.538
Soil PC2	−3.042–2.836	0.133	0.060
***Acer barbinerve*** ** (227)**			
DBH (cm)	1–3.7	1.640	1.5
Number of stems in a shrub	1–14	6.366	6
Basal area (m^2^) of overstory neighbors within 20 m	2.298–4.140	3.247	3.247
Basal area (m^2^) of understory neighbors within 5 m	0.012–0.062	0.031	0.029
Soil PC1	−1.991–3.340	0.781	1.068
Soil PC2	−1.595–2.430	0.400	0.208

Here we present only results for understory neighbors at 5 m radius and overstory neighbors at 20 m radius because GLMMs under these scales were the most likely models based on AIC and BIC for most cases when testing separately (Table S2 and S3 in File S1). We evaluated spatial autocorrelation in the residuals of the GLMMs (Figure S1 in File S1), and variograms revealed that the models adequately accounted for spatial autocorrelation.

All analyses were carried out in the statistical environment R (version 2.14.2), using the packages ‘geoR’ [30], ‘languageR’ [31], ‘lme4’ [32] and ‘sqldf’ [33].

## Results

### Effects of dbh and stem number in a shrub on stem growth and survival

**Figure 2:**
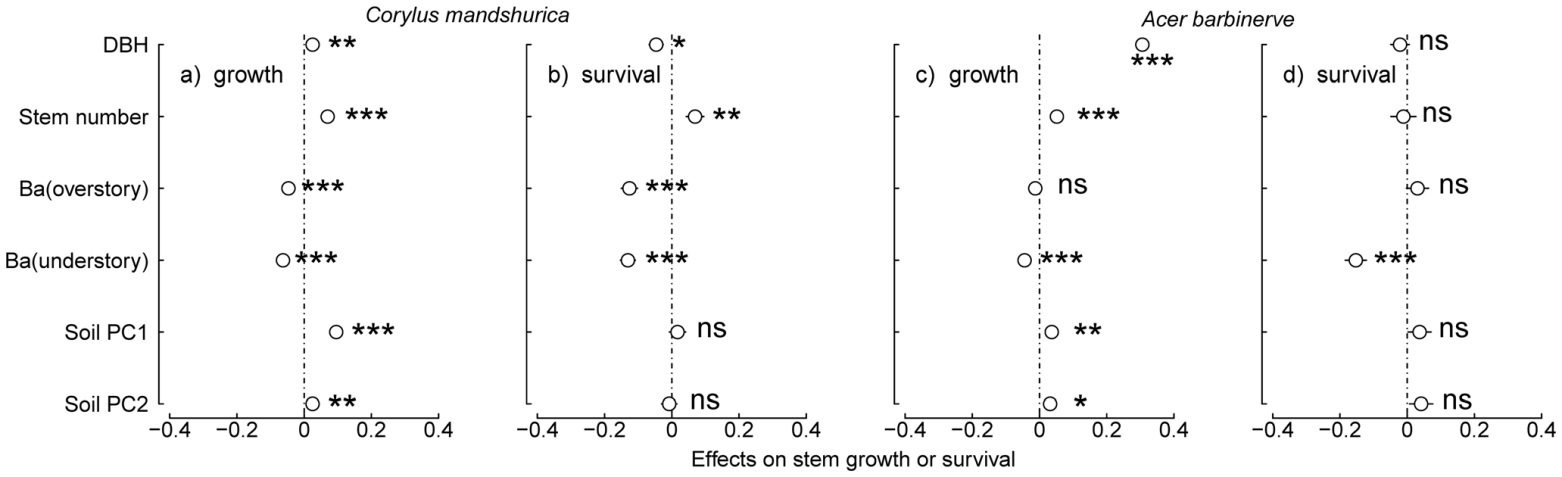
Effects of dbh, stem number in a shrub, neighborhood and soil variables on stem growth or survival of *C. mandshurica* and *A. barbinerve* in the old growth forest plot. *Circles* show the coefficient estimate for each parameter, with 2S.E. indicated by *horizontal lines*. “Ba” represents basal area. Asterisks represent the probability that the estimates are not different from zero: *P<0.05, **P<0.01, ***P<0.001, ns not significant (*P*>0.05).

**Figure 3:**
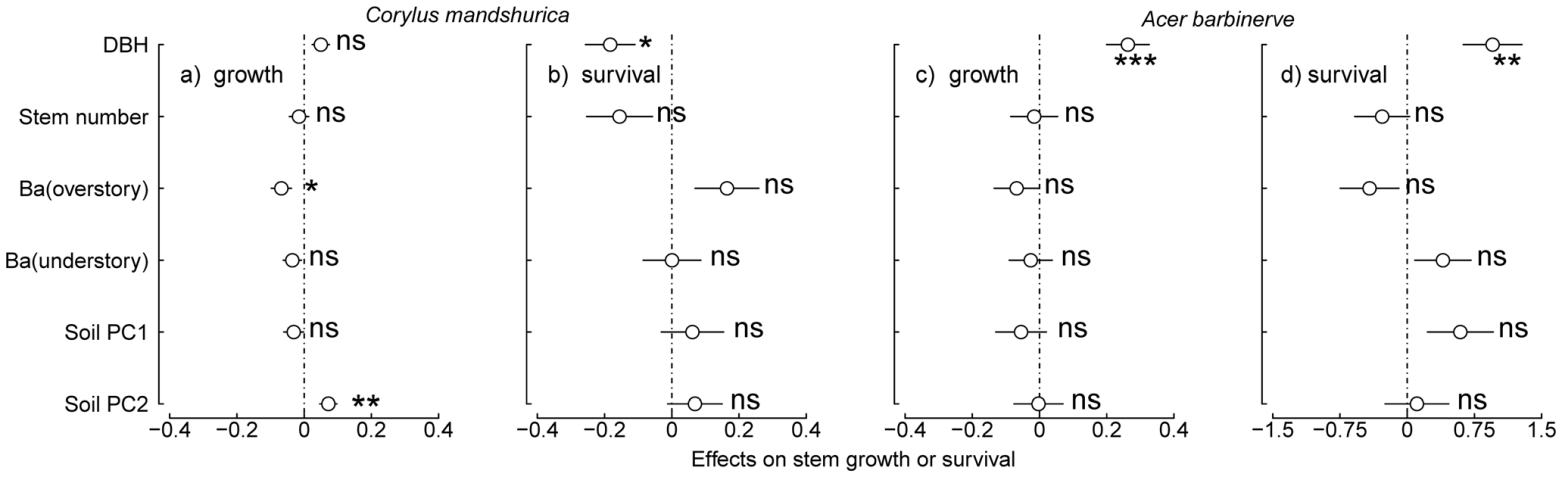
Effects of dbh, stem number in a shrub, neighborhood and soil variables on stem growth or survival of *C. mandshurica* and *A. barbinerve* in the secondary forest plot. *Circles* show coefficient estimate for each parameter, with 2S.E. indicated by *horizontal lines*. See Figure legend 2 for Ba, asterisks and ns.

Stem dbh had a strong negative influence on the survival of *C. mandshurica* in the two forests (Figure 2 and 3), such that larger stems had significantly higher mortality in the two forests. For example, stems of *C. mandshurica* of 1 cm dbh had probability of surviving of 0.736±0.002 in the old growth forest and 0.800±0.009 in the secondary forest. This probability declined to 0.696±0.014 and 0.522±0.064 for 3 cm dbh respectively. The effect of stem dbh was small but positive on the growth of *C. mandshurica* in the old growth forest. Stems of 1 cm dbh grew on average 0.200±0.005 cm^2^·yr^−1^ in the old growth forest, and this increased to 0.256±0.051 cm^2^·yr^−1^ for stems of 3 cm dbh.

Similarly, larger stems of *A. barbinerve* had faster growth in the two forests (for example, growth increased from 0.204±0.011 and 0.240±0.036 cm^2^·yr^−1^ at 1 cm dbh to 0.421±0.035 and 1.099 (only one stem) cm^2^·yr^−1^ at 3 cm dbh) (Figure 2 and 3). While in the secondary forest larger stems had a higher survival (increased from 0.821±0.050 to 0.939±0.000, at 1 and 3 cm dbh, respectively) (Figure 3).

The number of stems in an individual positively influenced growth and survival of *C. mandshurica* in the old growth forest (Growth: Effect±S.E. = 0.070±0.011, *P* = 5.45e-10; Survival: Effect±S.E. = 0.069±0.026, *P* = 0.008), but did not influence in the secondary forest (Figure 2 and 3). For example, average growth of stems of *C. mandshurica* in the old growth forest increased from 0.180±0.003 cm^2^·yr^−1^ in shrubs with only one stem, to 0.209±0.003 cm^2^·yr^−1^ in shrubs with two or more stems. Similarly, probability of survival of *C. mandshurica* in the old growth forest increased a very small but significant amount, going from 0.711±0.001 in shrubs with only one stem to 0.724±0.001 in shrubs with two or more stems. Moreover, in the old growth forest, there was a significant interaction between stem number and basal area of overstory neighbors in the survival of *C. mandshurica* (Effect±S.E. = 0.066±0.027, *P* = 0.014; not shown). This interaction suggests that the positive effect of multiple stems on survival was stronger when there were larger trees in the vicinity. The number of stems in a shrub also positively influenced the growth of *A. barbinerve* in the old growth forest (Effect±S.E. = 0.051±0.014, *P* = 0.0003), but not in the secondary forest, nor did it influence the survival of *A. barbinerve* in either of the two forest plots.

### Effects of neighborhood variables and soil variables in old growth and secondary forests

Consistent with our expectations, the effects of neighborhood variables and soil variables in the old growth forest were more apparent. For instance, in the old growth forest, basal area of overstory trees showed significant negative effects on growth and survival of *C. mandshurica*, such that both decreased when there were more, larger neighbors (Figure 2). This effect of overstory neighbors was not seen for *A. barbinerve* (Figure 2). However, basal area of understory neighbors had significant negative effects for both species (Figure 2). In contrast, in the secondary forest neighborhood variables had little effect on growth or survival of both species, with the exception of a negative effect of basal area of overstory trees for *C. mandshurica* (Figure 3). Growth of the two species in the old growth forest was influenced by soil variables (PC1 and PC2, Figure 2), suggesting that better soil nutrients lead to better growth. In contrast, in the secondary forest only soil PC2 had a positive effect on stem growth of *C. mandshurica* (Figure 3).

## Discussion

Our study showed differences in shrub dynamics between the two species and among the two forest sites.

We found that stems of *C. mandshurica* generally had decreased survival as they grew bigger. As older, larger stems probably have better resistance to physical damage and better access to light, decreasing survival can only be explained by stem senescence. We interpret this to mean that established shrubs of this species might shift their investment from surviving long lived stems to producing new stems. This same behavior was not found for *A. barvinerve*. On the contrary, larger stems of *A. barvinerve* in the secondary forest showed higher, not lower survival. While we cannot explain this with certainty, one possibility is that this population in the secondary forest is much younger. Indeed this species was much less abundant in this plot (45.4 stems·ha^−1^ vs. 408.64 stems·ha^−1^ in the old forest; Table 1), and stems were much smaller than in the old forest (max dbh of 3.7 cm vs. 13.2 cm in the old forest; Table 1). Hence, it is possible that most of the stems we captured were still growing and not senescing. *C. mandshurica* also had lower population density and smaller stems in the secondary forest, but this was not as marked as for *A. barvinerve* (Table 1).

Larger stems had higher basal area growth for *A. barvinerve* in the two forests, and also for *C. mandshurica* in the old growth forest. Similar results were found in recent studies of tree growth [34], [35], suggesting that larger stems had more biomass production than smaller ones. This result does not contradict the survival trends. As noted by previous research [12], size affects resource acquisition, as large individuals can obtain a disproportionately larger amount of the resources. Because stems require less diameter growth to produce the same biomass over an increasingly large area, basal area growth may be constant or increased, while diameter growth declines with stem size.

Multi-stemmed growth form was advantageous in some cases. The presence of multiple stems increased the growth of both species and survival of *C. mandshurica* in the old growth forest. Although the stems within multi-stemmed shrubs have more competition, shrubs with more stems have better resource acquisition, which may benefit stem performance. In multi-stemmed plants, the storage of carbohydrates is generally shared among stems [36], and this may be an effective strategy to buffer plants against stresses and improve survival [37] or growth. However, the advantage of multiple stems was not found in the secondary forest. While this study was unreplicated for stand age, and we can draw no firm conclusions. We hypothesize that there might be stronger competition from local neighbors in the old growth relative to the secondary forest. This conclusion is supported by the interaction between stem number and basal area of overstory neighbors in the survival of *C. mandshurica*. This interaction suggests that the effect of multiple stems is more important in areas of the forest with larger trees, that are probably also darker and more resource-limited. Also, random small-scale gap formation is more common in the old growth forest, and more patchily distributed light levels may be more favorable to multi-stemmed individuals [38].

There were considerable differences between the two species in the effects of overstory neighborhood basal area in the old growth forest. In fact, denser overstory neighborhoods significantly reduced stem growth and survival of *C. mandshurica* in the old growth forest but had little effect on *A. barbinerve*. Also, *A. barbinerve* performed better than *C. mandshurica* in the old growth forest (average of growth rate: 0.280±0.004 cm^2^·yr^−1^ vs. 0.198±0.002 cm^2^·yr^−1^; average probability of survival: 0.803±0.001 vs.0.721±0.001). Since higher basal area of neighbors indicates the presence of larger trees that may produce more shading, these results suggest that *A. barbinerve* may be better adapted to the understory environment in the old growth forest, while *C. mandshurica* is probably more sensitive to suppression with a smaller tolerance for shading. Consistent with previous studies [39]–[41], *C. mandshurica*, the less shade-tolerant species, also had higher mortality rates in these two forests.

While our data only provides limited information regarding the differences between forests, it was clear that shrub dynamics were different in the two forests. For example, the performance of the two species was clearly influenced by basal area of overstory or understory neighbors in the old growth forest, but not the secondary forest. The old growth forest in our study is characterized by overstory trees with larger diameter and greater basal area relative to the secondary forest (Table 1), which is consistent with previous research [42]. Because of crowding and shading from neighboring large trees, there is less available space in the understory of the old growth than the secondary forest. This may explain why the density of the local neighborhood was more important, and why both our study species had lower percent of surviving stems in the old growth forest relative to the secondary forest. The content of organic matter, total phosphorus and total potassium were significantly higher in the old growth forest, suggesting that soil nutrients are more available in old growth than developing phase. This observation is consistent with previous research in temperate forests [20]. This difference in soil characteristics among forests may explain why both our study species grew more in the old forest and their growth was positively correlated with soil variables in the old growth but not in the secondary forest.

Overall, we found that multi-stemmed growth form was an advantageous trait in the understory for *C. mandshurica* and partly for *A. barbinerve* in the old growth forest. Also, overstory neighbors influenced stem growth and survival for *C. mandshurica* but not for *A. barbinerve* in the old growth forest, which may suggest differences in shade tolerance among these two species. Differences in shade-tolerance among species commonly found in the understory have been reported before [40], but our study improves on previous studies in that we compared their dynamics in two forests with different structure. In our study, the old growth forest is characterized by overstory trees with larger diameter and greater basal area, and also more available soil nutrients relative to the secondary forest. According to the succession theory, the diversity of the secondary forest is not as stable as the old growth forest [43]. There may be more stochastic processes affecting dynamics of the two multi-stemmed understory shrub species in the secondary forest. Hence, we found that the effects of neighborhood and soil variables on growth and survival were not as strong in the secondary forest as in the old forest.

Generally, shrub species have different growth strategies than tall tree species. Many shrub species have more stems through vegetative reproduction, while overstory tree species are able to grow up into the canopy layer and sprout less frequently. Some authors have suggested that multi-stemmed growth form can be beneficial to understory species [10], while others have suggested it can be detrimental to stem dynamics [11]. Here we show that the effects of multiple stems differ for different species. Furthermore, even within the same species the benefits might be facultative, differing among forests and neighborhood compositions.

## Supporting Information

File S1 **Supporting tables and figure.** Table S1. Soil variable loadings for the two PCAs. Table S2. AIC, △AIC, BIC and △BIC values of stem growth and survival models of the two species for understory neighbors across different scales. The most likely models are shown in bold (red). Table S3. AIC, △AIC, BIC and △BIC values of stem growth and survival models of the two species for overstory neighbors across different scales. The most likely models are shown in bold (red). Figure S1. Variograms illustrating the spatial autocorrelation in residuals of the GLMMs for stem growth and survival of the two species in the old growth and secondary forest plots.
(DOC)
