# Supplementary material for: Dynamics of Two Multi-Stemmed Understory Shrubs in Two Temperate Forests
Source: PLoS One. 2014 Jun 2;9(6):e98200. doi: 10.1371/journal.pone.0098200 (PMC4041805; doi:10.1371/journal.pone.0098200)
Supplement: File S1 — Supporting tables and figure. Table S1. Soil variable loadings for the two PCAs. Table S2. AIC, △AIC, BIC and △BIC values of stem growth and survival models of the two species for understory neighbors across different scales. The most likely models are shown in bold (red). Table S3. AIC, △AIC, BIC and △BIC values of stem growth and survival models of the two species for overstory neighbors across different scales. The most likely models are shown in bold (red). Figure S1. Variograms illustrating the spatial autocorrelation in residuals of the GLMMs for stem growth and survival of the two species in the old growth and secondary forest plots. (DOC) [file pone.0098200.s001.doc]

Table S1 Soil variable loadings on the two PCAs.

| Soil variable | PCA1 | PCA2 |
| --- | --- | --- |
| Old growth  Total nitrogen | 0.603 | 0.360 |
| Total phosphorus | 0.600 | 0.384 |
| Total potassium | -0.526 | 0.850 |
| Variation explained | 85.3% | 13.5% |
|  |  |  |
| Secondary |  |  |
| Total nitrogen | -0.653 | 0.345 |
| Total phosphorus | -0.688 | 0.104 |
| Total potassium | 0.318 | 0.933 |
| Variation explained | 60.8% | 31.0% |

Table S2 AIC, △AIC, BIC and △BIC values of stem growth and survival models of the two species for understory neighbors across different scales*.* The most likely models are shown in bold (red).

| Radius  Instances | 5m | | 10m | | 15m | | 20m | | 5m | | 10m | | 15m | | 20m | |
| --- | --- | --- | --- | --- | --- | --- | --- | --- | --- | --- | --- | --- | --- | --- | --- | --- |
|  | AIC | △_AIC_ | AIC | △_AIC_ | AIC | △_AIC_ | AIC | △_AIC_ | BIC | △_BIC_ | BIC | △_BIC_ | BIC | △_BIC_ | BIC | △_BIC_ |
| *Corylus mandshurica* |  |  |  |  |  |  |  |  |  |  |  |  |  |  |  |  |
| Growth |  |  |  |  |  |  |  |  |  |  |  |  |  |  |  |  |
| Old growth forest | **-11558.647** | **0** | -11552.214 | 6.433 | -11555.641 | 3.006 | -11553.064 | 5.583 | **-11530.052** | **0** | -11523.619 | 6.433 | -11527.046 | 3.006 | -11524.469 | 5.583 |
| Secondary forest | **-1281.888** | **0** | **-1281.557** | **0.331** | **-1281.482** | **0.406** | **-1281.497** | **0.391** | **-1262.805** | **0** | **-1262.474** | **0.331** | **-1262.398** | **0.407** | **-1262.413** | **0.392** |
| Survival |  |  |  |  |  |  |  |  |  |  |  |  |  |  |  |  |
| Old growth forest | 16120.956 | 9.082 | **16113.156** | **1.282** | **16112.366** | **0.492** | **16111.874** | **0** | 16143.487 | 9.082 | **16135.687** | **1.282** | **16134.897** | **0.492** | **16134.405** | **0** |
| Secondary forest | **1496.337** | **1.977** | **1494.360** | **0** | **1495.184** | **0.824** | **1495.703** | **1.343** | **1511.775** | **1.978** | **1509.797** | **0** | **1510.622** | **0.825** | **1511.141** | **1.344** |
|  |  |  |  |  |  |  |  |  |  |  |  |  |  |  |  |  |
| *Acer barbinerve* |  |  |  |  |  |  |  |  |  |  |  |  |  |  |  |  |
| Growth |  |  |  |  |  |  |  |  |  |  |  |  |  |  |  |  |
| Old growth forest | **-4206.135** | **0** | -4202.491 | 3.643 | -4197.465 | 8.670 | -4199.449 | 6.686 | **-4178.227** | **0** | -4174.583 | 3.643 | -4169.557 | 8.670 | -4171.541 | 6.686 |
| Secondary forest | -161.857 | 5.298 | -161.391 | 5.765 | -162.594 | 4.561 | **-167.156** | **0** | -148.624 | 5.298 | -148.158 | 5.765 | -149.361 | 4.561 | **-153.923** | **0** |
| Survival |  |  |  |  |  |  |  |  |  |  |  |  |  |  |  |  |
| Old growth forest | **10336.415** | **0** | 10343.663 | 7.248 | 10347.664 | 11.249 | 10354.372 | 17.957 | **10358.110** | **0** | 10365.358 | 7.248 | 10369.359 | 11.249 | 10376.067 | 17.957 |
| Secondary forest | **153.036** | **0.482** | **152.554** | **0** | **153.485** | **0.931** | **153.531** | **0.977** | **163.311** | **0.482** | **162.829** | **0** | **163.760** | **0.931** | **163.806** | **0.977** |

Table S3 AIC, △AIC, BIC and △BIC values of stem growth and survival models of the two species for overstory neighbors across different scales*.* The most likely models are shown in bold (red).

| Radius  Instances | 5m | | 10m | | 15m | | 20m | | 5m | | 10m | | 15m | | 20m | |
| --- | --- | --- | --- | --- | --- | --- | --- | --- | --- | --- | --- | --- | --- | --- | --- | --- |
|  | AIC | △_AIC_ | AIC | △_AIC_ | AIC | △_AIC_ | AIC | △_AIC_ | BIC | △_BIC_ | BIC | △_BIC_ | BIC | △_BIC_ | BIC | △_BIC_ |
| *Corylus mandshurica* |  |  |  |  |  |  |  |  |  |  |  |  |  |  |  |  |
| Growth |  |  |  |  |  |  |  |  |  |  |  |  |  |  |  |  |
| Old growth forest | -11559.205 | 26.601 | -11567.557 | 18.249 | **-11585.806** | **0** | **-11584.636** | **1.170** | -11530.609 | 26.601 | -11538.962 | 18.248 | **-11557.210** | **0** | **-11556.041** | **1.169** |
| Secondary forest | -1291.816 | 3.161 | -1290.356 | 4.621 | **-1294.977** | **0** | -1289.779 | 5.198 | -1272.733 | 3.161 | -1271.273 | 4.621 | **-1275.894** | **0** | -1270.696 | 5.198 |
| Survival |  |  |  |  |  |  |  |  |  |  |  |  |  |  |  |  |
| Old growth forest | 16146.161 | 22.990 | 16141.348 | 18.177 | 16135.584 | 12.413 | **16123.171** | **0** | 16168.692 | 22.990 | 16163.879 | 18.177 | 16158.115 | 12.413 | **16145.702** | **0** |
| Secondary forest | 1494.118 | 8.075 | **1487.170** | **1.127** | **1486.043** | **0** | **1487.295** | **1.252** | 1509.556 | 8.075 | **1502.608** | **1.127** | **1501.481** | **0** | **1502.733** | **1.252** |
|  |  |  |  |  |  |  |  |  |  |  |  |  |  |  |  |  |
| *Acer barbinerve* |  |  |  |  |  |  |  |  |  |  |  |  |  |  |  |  |
| Growth |  |  |  |  |  |  |  |  |  |  |  |  |  |  |  |  |
| Old growth forest | -4199.109 | 7.643 | **-4206.752** | **0** | -4201.710 | 5.042 | -4197.602 | 9.150 | -4171.201 | 7.643 | **-4178.844** | **0** | -4173.802 | 5.042 | -4169.694 | 9.150 |
| Secondary forest | **-163.456** | **0** | **-162.778** | **0.678** | **-161.915** | **1.542** | **-161.882** | **1.574** | **-150.223** | **0** | **-149.545** | **0.678** | **-148.681** | **1.542** | **-148.649** | **1.574** |
| Survival |  |  |  |  |  |  |  |  |  |  |  |  |  |  |  |  |
| Old growth forest | **10357.744** | **0** | 10360.050 | 2.306 | **10359.696** | **1.951** | **10359.732** | **1.987** | **10379.439** | **0** | 10381.745 | 2.306 | **10381.391** | **1.952** | **10381.427** | **1.988** |
| Secondary forest | **152.051** | **0** | **152.541** | **0.490** | **153.738** | **1.687** | **152.145** | **0.093** | **162.326** | **0** | **162.816** | **0.490** | **164.013** | **1.687** | **162.419** | **0.093** |

Figure S1 Variograms illustrating the spatial autocorrelation in residuals of the GLMMs for stem growth and survival of the two species in the old growth and secondary forest plots.
